# Long‐term follow‐up results of spine tumor treatment using high‐dose radiotherapy after 3‐dimensional‐printed vertebral bodies implantation

**DOI:** 10.1002/cam4.5867

**Published:** 2023-03-31

**Authors:** Yao Xiao, Yuxia Wang, Feng Wei, Hongqing Zhuang

**Affiliations:** ^1^ Department of Radiation Oncology Peking University Third Hospital Beijing China; ^2^ Orthopedic Department Peking University Third Hospital Beijing China

**Keywords:** 3D‐printed vertebral body implantation, high‐dose radiotherapy (HDRT), postoperative radiotherapy (PORT), robotic radiosurgery, spinal tumors

## Abstract

**Objective:**

To investigate the long‐term safety and efficacy of high‐dose radiotherapy after 3D‐printed vertebral body implantation in the treatment of spinal tumors.

**Methods:**

Thirty‐three participants were recruited between July 2017 and August 2019. 3D‐printed vertebral bodies were implanted in each participant, followed by postoperative robotic stereotactic radiosurgery at a dose of 35–40 Gy/5f. The tolerance of the 3D‐printed vertebral body and the participant to the high‐dose radiotherapy were evaluated. In addition, the local control of tumor and the local progression‐free survival of the study participants following 3D‐printed vertebral body implantation and high‐dose radiotherapy were measured as indexes of effectiveness.

**Results:**

Of the 33 participants included in the study, 30, including three participants (10%) with esophagitis of grade 3 or above and two participants (6.7%) with advanced radiation nerve injury, successfully underwent postoperative high‐dose radiotherapy. The median follow‐up was 26.7 months, and IQR was 15.9 months. Most participants had primary bone tumors with 27 cases (81.8%), and the rest had bone metastases in six cases (18.2%). After high‐dose radiotherapy, the 3D‐printed vertebrae maintained good vertebral stability and exhibited histocompatibility, without implant fractures. The local control rates were 100%, 88%, and 85% 6 months, 1 year, and 2 years after high‐dose radiotherapy, respectively. Tumors recurred in four participants (12.1%) during the follow‐up period. The median local progression‐free survival after treatment was 25.7 months, with a range of 9.6–33.0 months.

**Conclusion:**

High‐dose radiotherapy for spinal tumors after 3D‐printed vertebral body implantation is feasible, elicits low toxicity, and yields satisfactory tumor control.

## Graphical abstract

To investigate the long‐term safety and efficacy of high‐dose radiotherapy after 3D‐printed vertebral body implantation in the treatment of spinal tumors. After high‐dose radiotherapy, the 3D‐printed vertebrae maintained good vertebral stability and exhibited histocompatibility, without implant fractures. The local control rates were 100%, 88%, and 85% 6 months, 1 year, and 2 years after high‐dose radiotherapy, respectively. Tumors recurred in four participants (12.1%) during the follow‐up period. The median local progression‐free survival after treatment was 25.7 months. High‐dose radiotherapy for spinal tumors after 3D‐printed vertebral body implantation is feasible, elicits low toxicity and yields satisfactory tumor control.

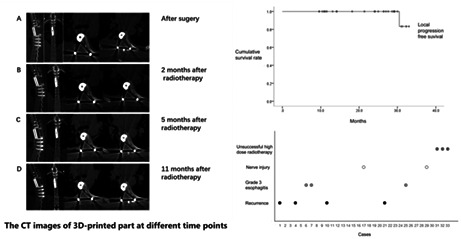

## 1 INTRODUCTION

The incidence of pain caused by bone tumors is very high. As one of the treatment methods, radiotherapy has been unanimously affirmed in its pain relief effect. Compared with standard multifraction radiotherapy (MFRT), SBRT has the characteristics of large single dose and less fractionation times, which is more advantageous in relieving bone pain.^1^, ^2^ However, the adverse reaction after palliative radiotherapy is still an urgent problem to be solved, such as vertebral compression fracture^3^, ^4^ and pain flare.^5^, ^6^ Therefore, comprehensive treatments, such as postoperative radiotherapy, are also needed to effectively treat spinal tumors.^7^, ^8^ 3D‐printed vertebral body implantation has significantly enhanced the treatment of spinal tumors, offering advantages of stability and histocompatibility compared with traditional implants.^9^, ^10^, ^11^ Theoretically, 3D‐printed vertebral body implants are more compatible with postoperative high‐dose radiotherapy than traditional implants. However, the tolerance to high‐dose radiotherapy after 3D‐printed vertebral body implantation and the effectiveness of this combination of therapies have not been studied in detail. These are the key issues of the final effect of spinal tumors, and also the ultimate test of the final effect of 3D printing technology, which has an important impact on clinical practice. The Peking University Third Hospital is the first medical facility in the world to treat spinal tumors using 3D‐printed vertebral body implants in combination with postoperative radiotherapy using the CyberKnife robotic radiosurgery system in patients for which it is indicated. In this study, this treatment protocol was applied to 33 participants, and the outcomes over long‐term follow‐up were investigated to observe the implementation of this technique and evaluate its curative effect as a clinical reference.

## 2 MATERIALS AND METHODS

### 2.1 Study protocol

**FIGURE 1:**
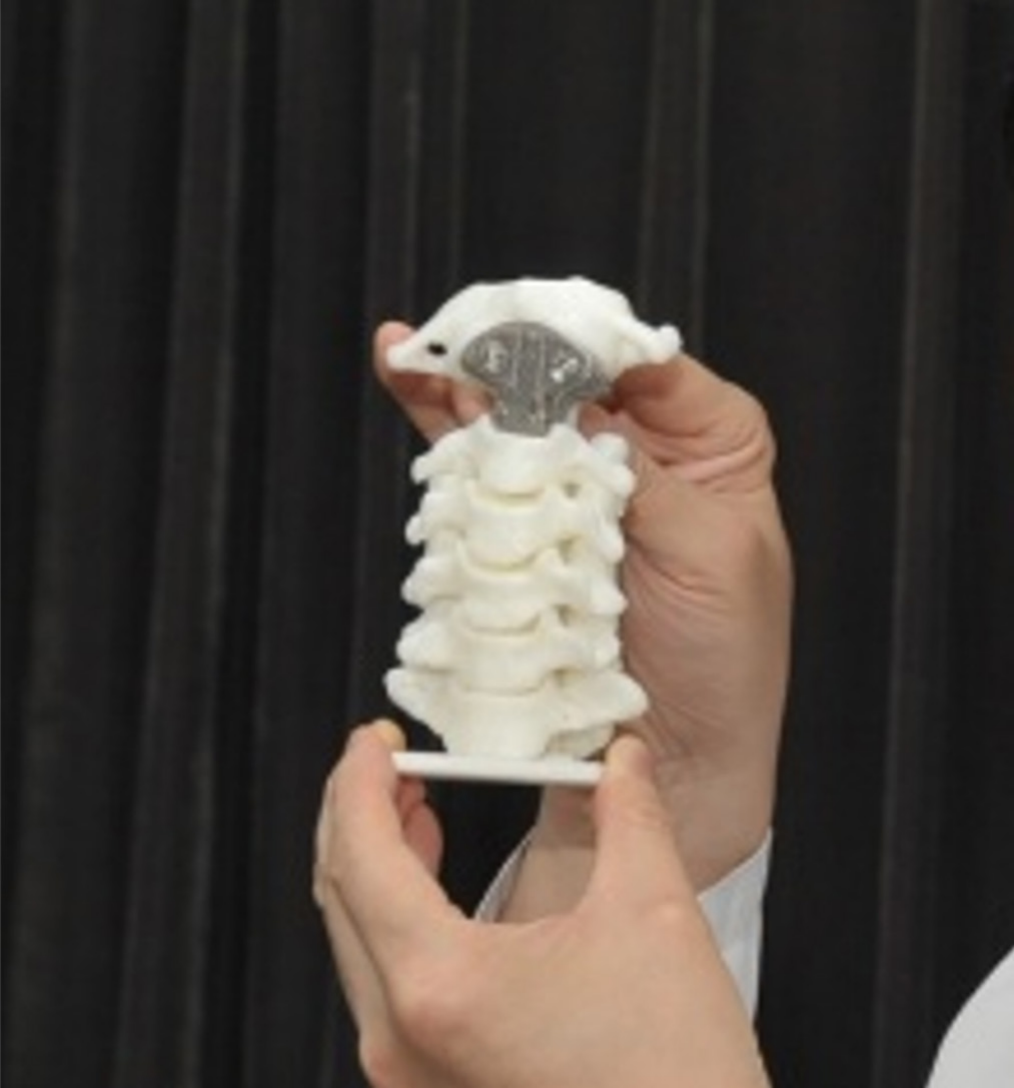
The picture of real 3D‐printed vertebral body.

As shown in Table 1, between 2017 and 2019, 3D‐printed vertebral bodies were implanted in a total of 33 participants with primary and metastatic spine tumors. Before surgery, all patients underwent a thin‐layer computed tomography (CT) and transmitted the CT data to a 3D‐printed implant supplier. The engineer then reconstructed the 3D model by software. After identifying the location and extent of spinal lesions, we quantified their structure and bone destruction. After that, according to the surgical design of the surgeon, the artificial vertebral body was designed. Once the design was complete, the data would be uploaded to the printing computer. Titanium alloy powder as raw material, with ultra‐high pressure electrode wire as the energy source. The implants are forged layer by layer using 3D printing technology from electron beam melting (Aikang, Beijing)^12^ (Figure 1). The mechanical properties and biocompatibility of the above materials have been tested and approved.^11^

**TABLE 1:** Baseline of patient and treatment characteristics.

Characteristic	Value(cases/percent)
Cases	33
Gender (cases/percent)	
Male	21/63.6
Female	12/36.4
Age (year)	
Range	18–74
Media	46
Primary or metastases (cases/percent)	
Primary	27/81.9
Metastases	6/18.1
Lesion site (cases/percent)	
Cervical vertebra	13/39.4
Thoracic vertebra	11/33.3
Lumbar vertebra	9/27.3
Pathology (cases/percent)	
Chordoma	9/27.3
Leiomyosarcoma	1/3.0
Chondrosarcoma	4/12.1
Rhabdomyosarcoma	1/3.0
Osteosarcoma	2/6.1
Solitary fibroma	1/3.0
Osteoblastoma	3/9.1
Solitary plasmacytoma	1/3.0
Giant‐cell tumor of bone	4/12.1
Ewing sarcoma	1/3.0
Metastases	6/18.2
Radiotherapy reason (cases/percent)	
Postoperative residual	11/33.3
High‐risk resection^a^	19/57.6
Postoperative recurrence	3/9.1
Dose (cGy)	
Range	3500–4000
Media	3500
Fraction	
Range	5–5
Media	5

^a^ High‐risk resection refers to trans‐tumor resection and high risk of cut edge.

Most of the patients who have completed postoperative radiotherapy had primary bone tumors with 24 cases (80%), and the rest had bone metastases in six cases (20%). Most cases staged by Enneking staging system. The cases included 7 chordomas (IIB, high grade, with six cases in G2 and one case in G3), 4 chondrosarcomas (IIB, high grade, G2), 1 leiomyosarcoma (IIB, high grade, G2), 1 rhabdomyosarcoma (IIB, high grade, G2), 2 osteosarcomas (IIB, high grade, G2), 1 solitary fibroma (IIB, high grade, G2), 3 osteoblastomas (IIB, high grade, G2), 3 giant‐cell tumor of bone (IIB, high grade, G2), and 1 ewing sarcoma (IIB, high grade, G3). The others included one solitary plasmacytoma (IIIA, by Durie‐Salmon staging system), and 6 metastases (IV, by AJCC 8th staging system, of which primary diseases include liver cancer, kidney cancer, thyroid cancer, and lung cancer).

The inclusion criteria were as follows: (1) prior diagnosis of a spinal tumor; (2) presence of residual after 3D‐printed vertebral body implantation or high risk of recurrence; (3) ability to tolerate high‐dose radiotherapy; and (4) initial radiotherapy. The exclusion criteria were as follows: (1) previous exposure to early‐stage radiotherapy (i.e., no reirradiation was performed); (2) inability to tolerate high‐dose radiotherapy due to comorbidities; (3) inability to lie down flat due to severe pain leading, inhibiting the inability to receive radiotherapy. This study was approved by the Ethics Committee of the Peking University Third Hospital and carried out under its supervision. Informed consent was obtained from all the participants, and the study protocol complied with all ethical guidelines.

### 2.2 Postoperative radiotherapy

**FIGURE 2:**
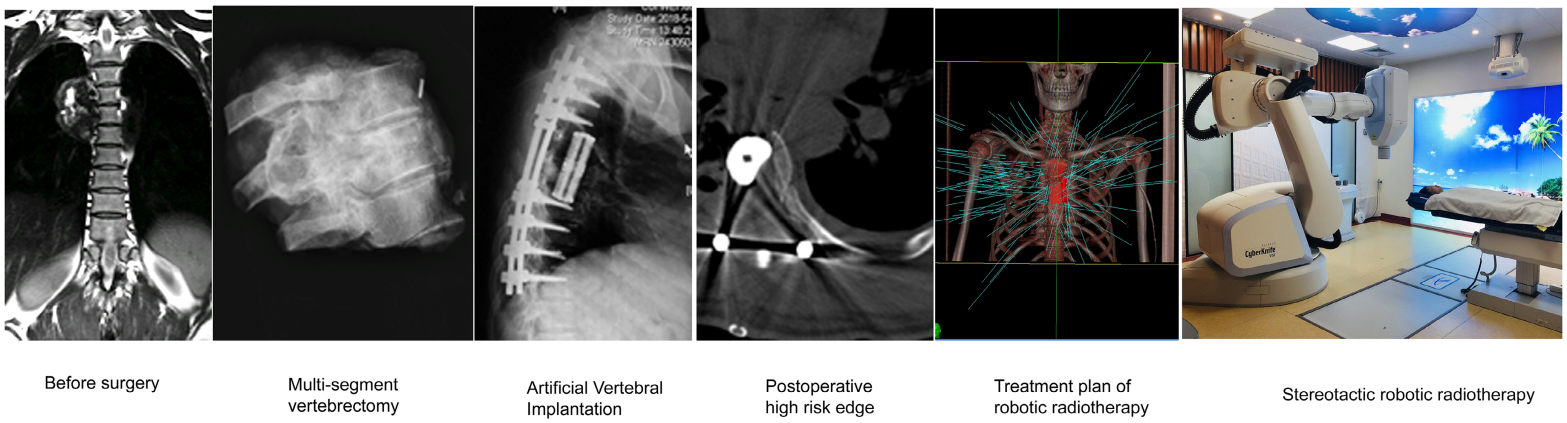
Flowchart of 3D‐printed vertebral body implantation combined with postoperative high‐dose radiotherapy.

**FIGURE 3:**
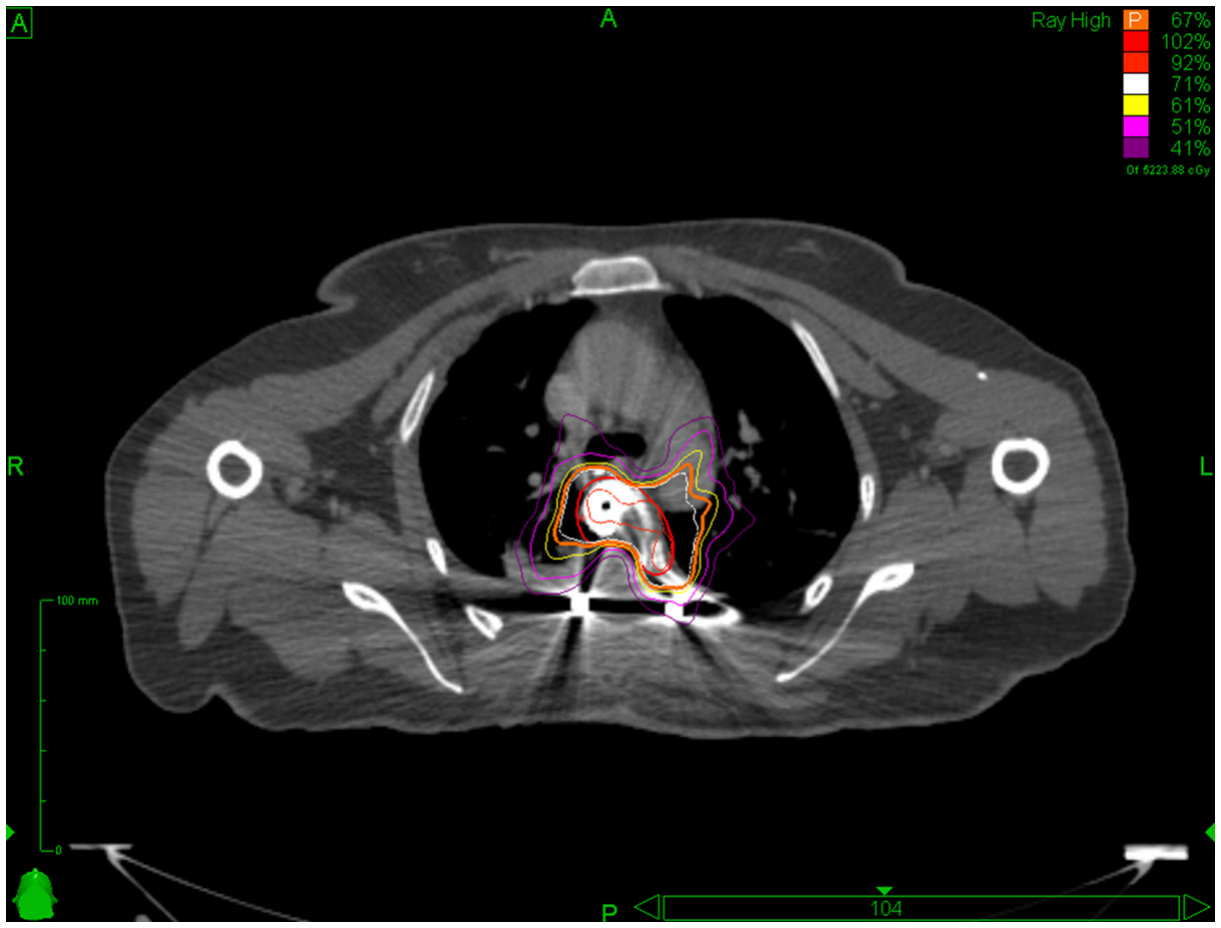
A plan of high‐dose radiotherapy treatment.

Postoperative radiotherapy was conducted with the CyberKnife robotic radiosurgery system (Iris Cyberknife).^13^, ^14^, ^15^ Postoperative magnetic resonance imaging (MRI) combined with computed tomography (CT) was used to map the target area and identify the involved organs. Enhanced MRI was conducted with a T1W1‐enhanced sequence to determine the range of the lesion and a T2W1 sequence to map the spinal cord. Thus, the target area for the treatment included the tumor‐involved regions identified preoperatively and the residual tumor areas and areas at high risk of recurrence identified postoperatively.^16^ The Multiplan 4.6 system was used to make the treatment plan. Postoperative stereotactic radiotherapy treatment was performed with a radiotherapy dose of 35–40 Gy/5f^17^, ^18^, ^19^, ^20^, ^21^, ^22^ administered by nonisocentric, noncoplanar irradiation. The Xsight Spine tracking system and a bed system with 3‐axis motion were used to determine the focus position and control the position of the participant, respectively. A schematic diagram of the implementation of high‐dose radiotherapy is shown in Figure 2. And a high‐dose radiotherapy treatment plan is shown in Figure 3.

### 2.3 Follow‐up and evaluation metrics

Routine reexaminations were conducted after stereotactic radiotherapy using spinal MRI, spinal CT, bone scanning, and X‐ray. The first reexamination was carried out 2 months after radiotherapy and every 3–4 months thereafter to evaluate the curative effect. At least four reexaminations were performed in the first year after radiotherapy, and the interval between reexaminations was no more than 6 months. The tolerance of the participants to postoperative high‐dose radiotherapy, the toxicity of the high‐dose radiotherapy, and tumor progression and recurrence were evaluated. Adverse reactions were observed according to CTCAE v4.0. The effect of the implant on the local tumor control rate was analyzed by bivariate correlation analysis, and the local progression‐free survival was analyzed by the Kaplan–Meier method. The statistical analyses were performed using SPSS23.0 software, and *p* < 0 0.05 was considered statistically significant.

## 3 RESULTS

### 3.1 Whether patients could perform high‐dose postoperative radiotherapy?

**FIGURE 4:**
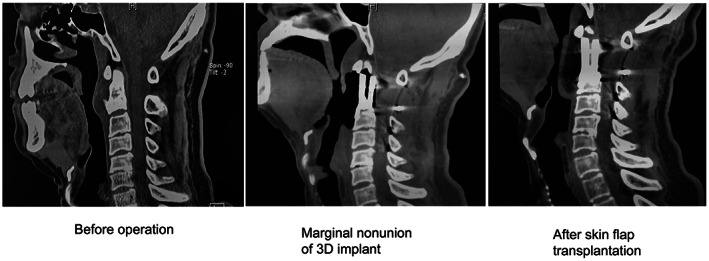
A case with poor postoperative healing after 3D‐printed vertebral body implantation, in which high‐dose radiotherapy could not be administered. Left: primary tumor on the anterior edge of the C2 vertebral body; middle: absence of soft tissue on the anterior edge of the 3D‐printed vertebral body after implantation; right: flap image of the anterior edge of the C2 vertebral body after flap transplantation.

Among the participants in this study, 30 successfully completed the postoperative high‐dose radiotherapy. The remaining three participants (9.1%) were not given postoperative high‐dose radiotherapy within 1 month after the operation because of comorbidities. All these patients had cervical spinal tumors, which caused problems with the growth of the oral and pharyngeal mucosa. As a result of this condition, the 3D‐printed vertebral body could not fit properly into the anatomy, allowing gas into the space between the 3D‐printed vertebral body and the surrounding tissue, which made it unsafe to administer high‐dose radiotherapy (Figure 4). In two participants, the ventral ulcer surface of the oral and pharyngeal mucosa did not heal after the operation, and thus pharyngeal flap transplantation was necessary; however, high‐dose radiotherapy could not be performed because of the limited tolerance of the flap. The third participant did not undergo high‐intensity radiotherapy because of possible radiation damage to the mucous membrane.

### 3.2 Tolerance to postoperative high‐dose radiotherapy

**FIGURE 5:**
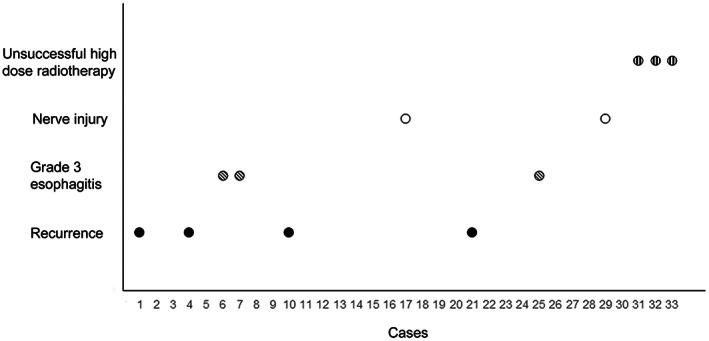
Scatter plot showing the adverse events, unsuccessful radiotherapy, and recurrence in all study participants. No participants exhibited both histocompatibility and vertebral implant instability issues.

The participants' tolerances to high‐dose radiotherapy were evaluated in term of the occurrence of the acute adverse events during radiotherapy including nausea, vomiting, burst pain, esophagitis, and stomatitis (Figure 5). Most adverse events were grade 1–2 reactions; there were only three cases of grade 3 esophagitis (3/30, 10%) that were improved after symptomatic treatment. During long‐term follow‐up, nerve injuries were observed in two participants (2/30, 6.7%), one involving the brachial plexus and the other involving the cervical nerve root.

**FIGURE 6:**
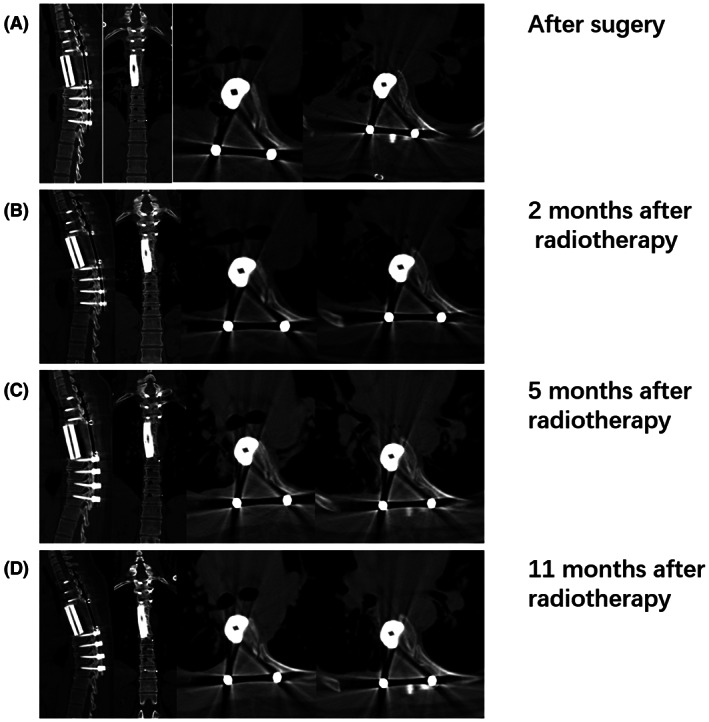
A typical patient who undergoes 3D‐printed vertebral implantation combined with high‐dose radiotherapy had CT images of 3D‐printed part at different time points.

The tolerance of the 3D‐printed vertebral body implant to high‐dose radiotherapy was evaluated in terms of the histocompatibility and vertebral stability. The histocompatibility and stability of the vertebral body were acceptable in all participants receiving radiotherapy (30/30, 100%), and no fractures in the vertebral body implants were observed. Pictures to compare the shape and geometry of the 3D‐printed parts at various time points is shown in Figure 6. The good histocompatibility and stability of vertebral implant demonstrate that the 3D‐printed vertebral body tolerated the high‐dose radiotherapy well, which provides evidence in support of an important advantage of 3D printing vertebral body implants.

### 3.3 Local control of postoperative radiotherapy

The local control rates of 6 months, 1 year, and 2 years after high‐dose radiotherapy were 100%, 88%, and 85%, respectively, and a total of four participants (12.1%) had recurrence during the follow‐up period (Figure 5). These results indicate that 3D‐printed vertebral body implantation combined with high‐dose radiotherapy provides good local tumor control.

Considering that artifacts are often observed in MRI after vertebral implantation, which hinder the mapping of the target area and spinal cord and limit the precision of treatment, the MRI artifacts in the different cases were evaluated. Results showed that postoperative recurrence was significantly correlated with presence of postoperative MRI artifacts (Pearson correlation coefficient = 0.65, *p* = 0.000). Therefore, it might be inferred the influence of postoperative artifacts on precious radiotherapy was a contributing factor leading to recurrence.

### 3.4 Local progression‐free survival of participants

**FIGURE 7:**
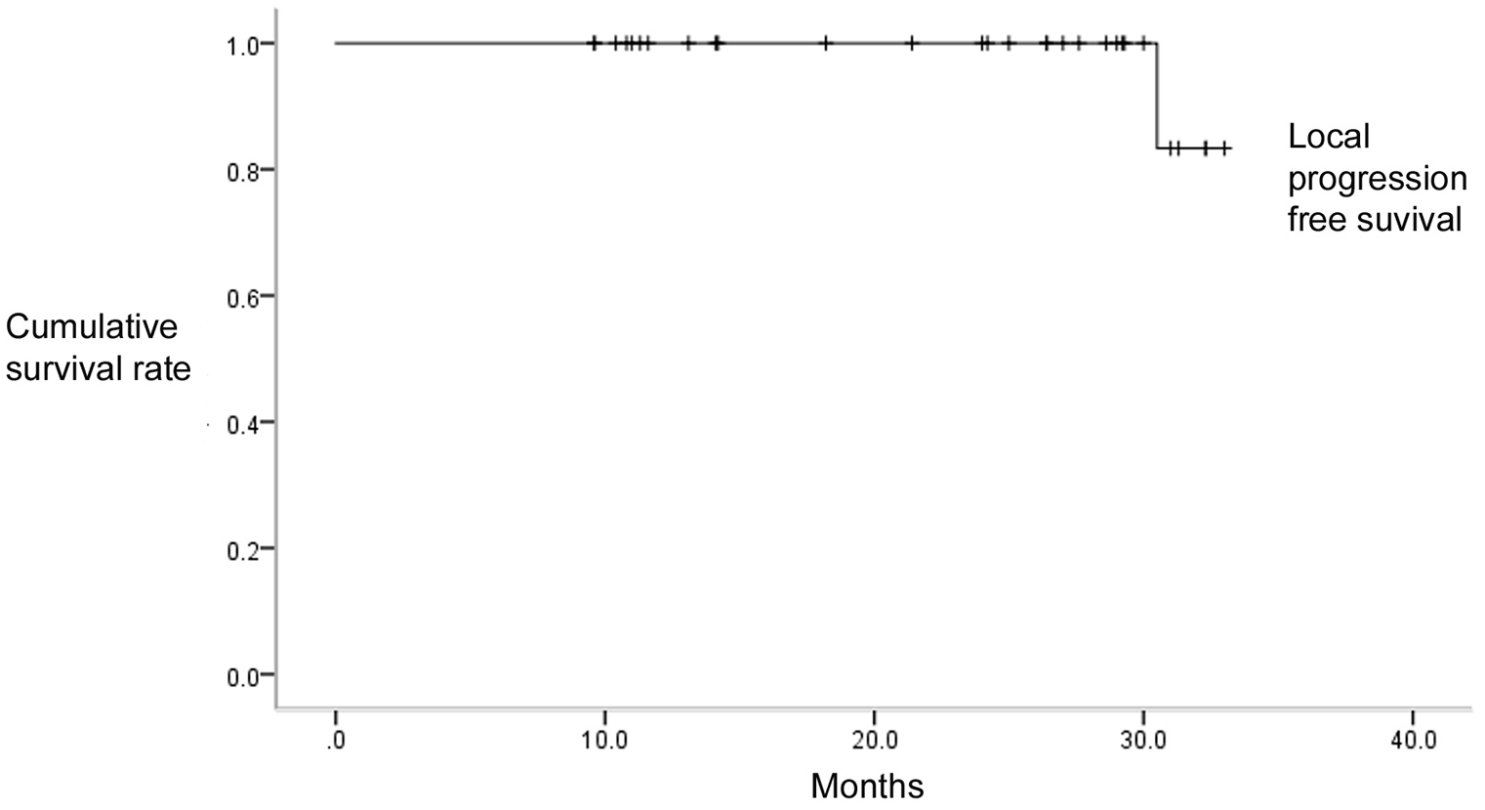
Local progression‐free survival curve of the subjects. The results showed that 3D‐printed vertebral body implantation combined with postoperative high‐dose radiotherapy resulted in good local control of cancer progression.

Kaplan–Meier survival analysis was conducted, and a survival curve was constructed (Figure 7). The local progression‐free survival after 3D‐printed vertebral body implantation combined with high‐dose radiotherapy was 9.6–33.0 months, with a median of 25.7 months.

## 4 DISCUSSION

The results of this study suggest that 3D‐printed vertebral body implants have good tolerance to high‐dose radiotherapy in the treatment of spinal tumors, exhibit good histocompatibility, and provide a good local tumor control effect. The 3D‐printed vertebral body implants were also found to be reliable over long‐term follow‐up. These findings imply that this approach may be revolutionary in the treatment of spinal tumors.

The observed benefits of the 3D‐printed vertebral body implant over long‐term follow‐up, including its good histocompatibility and robustness under high‐dose radiotherapy can be attributed to its design: Firstly, the implant was fabricated from a titanium alloy, which facilitates the growth of bone tissue.^23^ Secondly, the grid structure of the 3D‐printed vertebral body promotes better growth and binding of bone tissues compared with traditional implants.^24^, ^25^ Finally, the 3D‐printed vertebral body can be custom‐designed to better fit between the adjacent upper and lower vertebral bodies, which contributes to its histocompatibility and stability.^26^

However, the characteristics of the 3D‐printed implant also lead to artifacts on postoperative MRI scans. Such artifacts are an important problem because they hinder the mapping of the target area and involved organs, thereby confounding the high‐intensity precious radiotherapy and negatively affecting the precision and curative effect of postoperative radiotherapy. The development of novel 3D printing materials and more reasonable MRI sequences are the direction of continued efforts in future research.

This study is the first long‐term follow‐up of high‐dose radiotherapy after 3D‐printed vertebral body implantation. Our findings suggest that the 3D‐printed implant has excellent histocompatibility and provides excellent vertebral stability after high‐dose radiotherapy. These findings represent a breakthrough in clinical treatment to preserve patient function. However, the performance of 3D printing needs to be objectively evaluated after these conceptually promising initial findings. It should also be noted that artifacts caused by the 3D‐printed vertebral body implant somewhat hinder the subsequent tumor radiotherapy. This study provides a reference for better understanding 3D‐printed vertebral body implantation and postoperative high‐dose radiotherapy in the treatment of spinal tumors and provides new ideas for the further improvement of 3D‐printed vertebral body implants.

### 4.1 Limitations

This study was limited in that it was a single‐center study because 3D printing is not popular globally and needs to be compared with traditional surgical implant materials; similarly, postoperative precious stereotactic radiotherapy is a recently emerging technique in spinal tumor treatment, and the comparison with different radiotherapy techniques is needed.^1^, ^2^ Therefore, this combined treatment needs to be more popularized, and the conclusions of this study should be verified in multicenter studies.

## 5 CONCLUSIONS

In conclusion, our findings indicate that high‐dose radiotherapy following 3D‐printed vertebral body implantation is feasible. The 3D‐printed vertebral body implant still maintains good histocompatibility and stability after high‐dose radiotherapy, and the combination of these treatment techniques provides good local tumor control. The results of this study provide insight into the applicability of 3D printing technology in the removal of spinal tumors in combination with radiotherapy, including the demonstration of the long‐term reliability of 3D‐printed vertebral body implants following radiotherapy. Hence, 3D‐printed vertebral body implantation combined with postoperative high‐dose radiotherapy is expected to be a reliable new method for the treatment of spinal tumors.

## AUTHOR CONTRIBUTIONS

**Yao Xiao:** Conceptualization (equal); methodology (equal); writing – original draft (equal); writing – review and editing (equal). **Yuxia Wang:** Conceptualization (equal); formal analysis (equal); methodology (equal); writing – original draft (equal). **Feng Wei:** Conceptualization (equal); data curation (equal); methodology (equal); project administration (equal); supervision (equal). **Hongqing Zhuang:** Conceptualization (equal); data curation (equal); funding acquisition (equal); methodology (equal); writing – original draft (equal); writing – review and editing (equal).

## FUNDING INFORMATION

This research was funded by Clinical Cohort Construction Program of Peking University Third Hospital, BYSYDL2021009.

## ETHICS APPROVAL AND CONSENT TO PARTICIPATE

The full name and affiliation of the ethics committee that approved this study is Peking University Third Hospital Medical Science Research Ethics Committee. And the research ethics approval number is M2020259. The form of consent was written, and permission was obtained from each of the subjects to participate in the study.

## Data Availability

Data sharing is not applicable to this article as these patients need further follow up, and we will open the data on the clinical registration website when the study finished.
